# Prevalence and risk factors of gestational diabetes mellitus among pregnant women in northern Vietnam: a cross-sectional study

**DOI:** 10.1080/16549716.2025.2460339

**Published:** 2025-02-10

**Authors:** Hieu Minh Le, Thi Ai Nguyen, Dang Kien Nguyen, Ditte Søndergaard Linde, Ib Christian Bygbjerg, Jens Søndergaard, Thanh Duc Nguyen, Bay Quang Nguyen, Ngoc-Anh Thi Dang, Xuan Bai Nguyen, Dan W. Meyrowitsch, Christina A. Vinter, Tine M. Gammeltoft, Vibeke Rasch

**Affiliations:** a Department of Internal Medicine, Thai Binh University of Medicine and Pharmacy, Thai Binh, Vietnam; b Department of Internal Medicine, Hanoi Medical University, Hanoi, Vietnam; c Faculty of Public Health, Thai Binh University of Medicine and Pharmacy, Thai Binh, Vietnam; d Department of Gynaecology and Obstetrics, Thai Binh University of Medicine and Pharmacy, Thai Binh, Vietnam; e Department of Gynaecology and Obstetrics, Odense University Hospital, Odense, Denmark; f Department of Clinical Research, University of Southern Denmark, Odense, Denmark; g Department of Public Health, Global Health Section, University of Copenhagen, Copenhagen, Denmark; h Research Unit of General Practice, Department of Public Health, University of Southern Denmark, Odense, Denmark; i Department of Endocrinology, Bach Mai Hospital, Hanoi, Vietnam; j Department of Histology, Thai Binh University of Medicine and Pharmacy, Thai Binh, Vietnam; k Department of Anthropology, University of Copenhagen, Copenhagen, Denmark

**Keywords:** Family health, fertility treatment, gestational diabetes mellitus, pregnancy, risk factors, prenatal care

## Abstract

**Background:**

Gestational diabetes mellitus (GDM) increases adverse neonatal and maternal outcomes. Understanding the prevalence and risk factors of GDM is necessary to plan health care interventions and policy.

**Objective:**

To determine the prevalence and risk factors of GDM in Thai Binh, Vietnam.

**Methods:**

A cross-sectional study was conducted in two health facilities in Thai Binh, Vietnam, with the participation of 1,106 pregnant women. Women were recruited at their first antenatal care visit where face-to-face interviews about socioeconomic and reproductive factors were performed. A 2-hour 75 g oral glucose tolerance test was conducted at 24–28 weeks of gestation. GDM was diagnosed according to the World Health Organization 2013 criteria. Logistic regression analyses were used to assess the factors associated with GDM.

**Results:**

The prevalence rate of GDM was 27.1%. Multivariate logistic regression analysis showed maternal age from 25 to 34 (adjusted OR 2.0; 95%CI 1.3–2.9), maternal age ≥ 35 (adjusted OR 3.0; 95%CI 1.7–5.4), pregestational body mass index ≥ 23 (adjusted OR 1.6; 95%CI 1.1–2.3), family history of diabetes (adjusted OR 1.9; 95%CI 1.3–2.9), fertility treatment (adjusted OR 2.3; 95%CI 1.3–3.8), and previous GDM (adjusted OR 3.1; 95%CI 1.4–6.9) were associated with increased odds of GDM.

**Conclusions:**

More than one-fourth of pregnant women in Thai Binh, Vietnam, may have GDM. Advanced maternal age, high pregestational body mass index, family history of diabetes, and previous GDM were associated with increased risk of GDM. Additionally, fertility treatment appears to be strongly associated with an increased risk of GDM.

## PAPER CONTEXT

**Main findings:**

This study highlights the prevalence of gestational diabetes in semi-urban areas of Vietnam and its association with fertility treatment.

**Added knowledge:**

The prevalence of gestational diabetes is high, and fertility treatment is strongly associated with the increasing risk of gestational diabetes in semi-urban areas in lower-middle-income countries.

**Global health impact for policy and action:**

Intervention efforts to prevent gestational diabetes need to focus on women of reproductive age at high risk due to factors such as fertility treatment, obesity, advanced age, previous macrosomia, and previous gestational diabetes before pregnancy.

## Background

Gestational diabetes mellitus (GDM) is defined as glucose intolerance with onset or first recognition during pregnancy [1]. GDM not only increases adverse fetal outcomes such as macrosomia and large for gestational age but is also associated with adverse maternal outcomes, including increased risk of preeclampsia and caesarean section [2]. Further, GDM is associated with an increased risk of developing type 2 diabetes in both mother and child [3–5].

GDM is a global health issue with a 7–28% prevalence rate, varying by region, ethnicity, and diagnostic criteria [6]. Known risk factors of GDM include advanced age, multiparity, high pregestational body mass index (BMI), family history of diabetes, and lifestyle factors such as diet, and physical activity [7–9]. In multiparous women, previous macrosomia and previous GDM are also associated with an increased risk of recurrent GDM [8]. Recently, it has also been documented that fertility treatment is associated with an increased risk of GDM, with a meta-analysis showing that women who conceive after assisted reproductive technology have an increased risk of 1.5 of GDM [10]. Similarly, a register-based study conducted in Finland observed an increased risk of GDM, with a risk ratio of 1.3 among women who conceived after assisted reproductive technology compared to those who conceived spontaneously [11]. In addition, polycystic ovary syndrome (PCOS) is associated with an increased risk of GDM [12,13].

In Vietnam, the national guideline recommends universal GDM screening for all pregnant women between gestational weeks 24 and 28, following the World Health Organization 2013 criteria. However, data on the prevalence and risk factors of GDM in Vietnam are limited; studies have been restricted to the three metropolitan cities, with prevalence rates varying between 6% and 23% [14–17]. In Vietnam, BMI and maternal age have been reported as strong predictors of GDM. Conversely, previous macrosomia and previous GDM were not associated with GDM [14]. To date, there is a lack of knowledge about GDM in provincial areas. Therefore, we aimed to determine the prevalence of GDM and risk factors for GDM in a provincial area in northern Vietnam.

## Methods

### Study design and setting

A cross-sectional study was conducted in Thai Binh Province of northern Vietnam. Data were collected in two periods: from January to August 2023 and from January to February 2024. Thai Binh Province covers an area of 1584.6 km^2^. The population of the region is approximately 1,882,300, and it had a crude birth rate of 12.1 per 1000 people in 2023 [18]. Thai Binh Province includes one city and seven districts. Participants were recruited from two health facilities: Thai Binh Maternity Hospital and Kim Ngan Clinic in Thai Binh City. Thai Binh Maternity Hospital is the largest public maternity hospital in Thai Binh Province, where pregnant women can attend antenatal care and deliver, with approximately 11,000 deliveries per year. Kim Ngan Clinic is a private facility where pregnant women can only attend antenatal care, with approximately 8,000 cases per year.

### Participants

Inclusion criteria were: 1) Age 18 years or above; 2) Pregnancy <28 weeks; 3) Residing in Thai Binh Province; and 4) Voluntary participation after informed consent. Exclusion criteria were: 1) Pre-gestational diabetes; and 2) Severe chronic disease.

### Recruitment

Pregnant women were invited to participate in the study at their first antenatal care visit from 5 to 28 gestational weeks. After written informed consent was obtained, the women participated in a questionnaire interview and were scheduled for an Oral Glucose Tolerance Test (OGTT) in their 24th to 28th gestational weeks.

### Oral glucose tolerance test and diagnosis of gestational diabetes mellitus

All women were offered an OGTT, which was performed at Thai Binh Maternity Hospital and Kim Ngan Clinic. The procedure began with the women fasting overnight and attending the clinic in the morning. A venous blood sample was first taken. Subsequently, the women were offered a 75 g glucose solution, which they consumed within five minutes, while seated. Additional venous blood samples were collected at 1 and 2 hours post-ingestion. The sample tubes were centrifuged for 15–30 minutes to separate blood cells. Plasma glucose levels were measured using a glucose oxidase method (Analyzer; Model: BA400; Biosystems, Barcelona; Spain) at Thai Binh Maternity Hospital, and a hexokinase method (Cobas 4000; Model: c311; Roche, Mannheim, Germany) at Kim Ngan Clinic. GDM was diagnosed based on the World Health Organization 2013 criteria, which include fasting plasma glucose levels ≥5.1 mmol/l and <7.0 mmol/l, or 1-hour glucose ≥10.0 mmol/l, or 2-hour glucose ≥8.5 mmol/l and <11.1 mmol/l. Overt diabetes in pregnancy was diagnosed according to the World Health Organization 2013 criteria (fasting plasma glucose levels ≥7.0 mmol/l or 2-hour glucose ≥11.1 mmol/l) [1].

### Risk factor assessment

Self-reported pregestational weight was registered at the time of inclusion, and measurements of the women’s current height and weight were recorded. Additionally, the women’s weight was also measured at the OGTT visit. Pregestational BMI was calculated and classified according to the Asian-Pacific cutoffs: underweight (<18.50 kg/m^2^), normal weight (18.50–22.99 kg/m^2^), overweight (23.00–24.99 kg/m^2^), and obesity (≥25.00 kg/m^2^) [19]. Data on sociodemographics were collected at recruitment and included maternal age, marital status, educational level, occupation, living area, and economic status. Information about reproductive and GDM risk factors was also gathered. This included parity, history of abortion, history of stillbirth, history of premature birth, history of macrosomia, history of GDM, family history of diabetes, PCOS, fertility treatment, pregnancy-induced hypertension, and use of anti-depressive medication. Information regarding fertility treatment included ovulation induction, intrauterine insemination, in vitro fertilisation (IVF), and intracytoplasmic sperm injection. Finally, information about lifestyle characteristics such as alcohol consumption, exposure to smoke, and physical activity was also collected. Physical activity was assessed through women’s reports of the average number of hours spent on low- and high-intensity physical activities per week.

### Statistical analysis

Data were entered into REDcap and analyzed using the Statistical Package for Social Sciences version 22.0. Descriptive statistics were used to delineate sociodemographic and reproductive characteristics, as well as the prevalence of GDM among participants. The normal distribution of continuous variables was examined, and appropriate descriptive analyses were used. Variables with normal distribution were described using mean and standard deviation; median and interquartile range (IQR) were deployed for variables with non-normal distribution. Potential risk factors, including living area, maternal age, pregestational BMI, family history of diabetes, parity, PCOS, fertility treatment, use of anti-depressive medication, exposure to smoke, and levels of physical activity, were analysed. Logistic regression analyses were employed to assess factors associated with GDM, estimating odds ratio (OR) and 95% confidence interval (95%CI). Crude ORs and maternal age adjusted ORs (aORs) were calculated. Subsequently, adjustments were made for maternal age, pregestational BMI, family history of diabetes, parity, and fertility treatment. These factors were selected a priori based on the literature. Only variables with a 5% significance level in the unadjusted analysis were included in the adjusted analyses. An additional analysis among multiparous women explored the possible associations between obstetric risk factors (history of GDM, history of macrosomia, history of pregnancy-induced hypertension, and previous pregnancy outcomes) and GDM in the current pregnancy. Crude ORs and aORs along with 95%CIs were used to describe the associations between obstetric risk factors and GDM. Maternal age, pregestational BMI, family history of diabetes, parity, fertility treatment, history of GDM, and history of macrosomia were considered in the adjusted analysis.

### Ethics

The study was approved by the Ethics Council in Biomedical Research of Thai Binh University of Medicine and Pharmacy, Vietnam (IRB – VN01.009) on 1 December 2022 (Number of reference: 1325/HDDD). Data were collected as part of the VALID-II study (Clinicaltrials.gov: NCT05744856).

## Results

### Baseline characteristics of study participants

**Figure 1.**
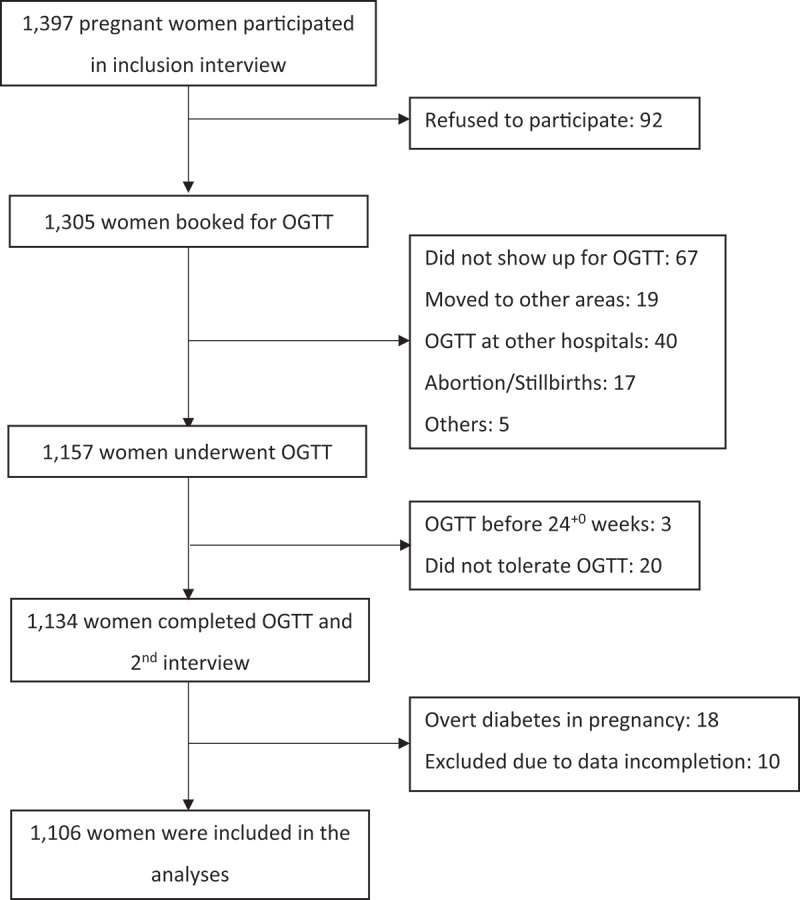
Flow chart of participants in the study.

Among 1,397 pregnant women included in the study, 1,305 (93.4%) agreed to participate and were scheduled for OGTT, while 92 (6.6%) declined (Figure 1). In all, 1,157 women underwent OGTT (82.8% of eligible women). However, 20 women did not tolerate the OGTT, three women had completed the test before 24 weeks of gestation, and 18 (1.6%) women were diagnosed with overt diabetes in pregnancy. These women were excluded from the analyses together with 10 women who had incomplete questionnaires. Thus, a total of 1,106 women were included in the analyses, of whom 311 (28.1%) were recruited from Thai Binh Maternity Hospital and 795 (71.9%) were from Kim Ngan Clinic.

OGTT is oral glucose tolerance test.

The baseline maternal characteristics are shown in Table 1. The median and IQR of the age of participants were 27 [24–31]. Approximately two-thirds (64.4%) were below 30 years of age. Five (0.5%) of the women were smokers, while one-third (32.5%) were exposed to passive smoking. In total, 16.4% of the women were overweight or obese, 645 (58.3%) were multiparous, one-fifth (20.2%) had delivered two times or more, and 68 (6.2%) of the women had conceived after fertility treatment in relation to the current pregnancy. In our study, among the 68 pregnant women who underwent fertility treatment, 3 (4.4%) used ovulation induction, 7 (10.3%) underwent intrauterine insemination, 52 (76.5%) utilized in vitro fertilization, and 6 (8.8%) were unable to recall the specific method used.

**Table 1.** Characteristics of study participants (*n* = 1,106).

Maternal characteristics	Number of participants (%) (*n* = 1,106)
Age, median (IQR)	27 (24–31)
≤24 25–29 30–34 35–39 ≥40	307 (27.8) 405 (36.6) 262 (23.7) 101 (9.1) 31 (2.8)
Living areas	
Urban Rural	433 (39.2) 673 (60.8)
Education level	
Primary school/Secondary school High school College/University and above	105 (9.5) 348 (31.5) 653 (59.0)
Occupation	
Unemployed/Student/No longer in job due to pregnancy Factory Worker/Farmer Government officer/Work for private company Small trade Homemaker Others	37 (3.3) 321 (29.0) 340 (30.7) 141 (12.8) 126 (11.4) 141 (12.8)
Marital status (Cohabiting)	
Living together Not living together Others	1,030 (93.1) 67 (6.1) 9 (0.8)
Economic status (Self-reported)	
Poor/Near poor Medium Wealthy	5 (0.5) 1,091 (98.6) 10 (0.9)
Daily mode of translation	
Bicycle/Electronic bicycle Motorbike Private car Public transport	46 (4.2) 969 (87.6) 84 (7.6) 7 (0.6)
Alcohol intake	66 (6.0)
Exposed to smoke Smoking Passive smoking Open-fire or charcoal cooking	373 (33.7) 5 (0.5) 360 (32.5) 20 (1.8)
Pregestational body mass index (kg/m^2^), median (IQR) Underweight <18.5 Normal 18.5–22.99 Overweight 23.0–24.99 Obesity ≥25.0	20.4 (18.9–22.1) 204 (18.4) 721 (65.2) 114 (10.3) 67 (6.1)
Weight gain per week (kg/week), median (IQR) ≤0.19 0.19–0.35 ≥0.35	0.27 (0.19–0.35) 268 (24.2) 563 (50.9) 275 (24.9)
Number of previous pregnancies	
0 1 2 3 and above	425 (38.4) 263 (23.8) 153 (13.8) 265 (24.0)
Parity	
Nulliparous 1 time 2 times and above	461 (41.7) 422 (38.1) 223 (20.2)
Gestational age at the time of oral glucose tolerance test	
24 25 26 27 plus	372 (33.7) 258 (23.3) 251 (22.7) 225 (20.3)

IQR is interquartile range.

### Prevalence of GDM

Of the 1,106 included women, 27.1% were diagnosed with GDM (Table 2). The median values and IQRs of fasting, 1-hour, and 2-hour glucose levels of these women were 4.60 (4.39–4.83), 7.94 (6.75–9.28), and 7.03 (6.19–8.07) mmol/l, respectively.

**Table 2.** Oral glucose tolerance test results (*n* = 1,106).

75 g oral glucose tolerance test	Number of participants (%) (n = 1,106)
Fasting plasma glucose (mmol/l), median (IQR) <5.10 5.10–6.99	4.60 (4.39–4.83) 1,012 (91.5) 94 (8.5)
1-hour plasma glucose (mmol/l), median (IQR) <10.00 ≥10.00	7.94 (6.75–9.28) 935 (84.5) 171 (15.5)
2-hour plasma glucose (mmol/l), median (IQR) <8.50 8.50–11.09	7.03 (6.19–8.07) 910 (82.3) 196 (17.7)
Result of oral glucose tolerance test	
Normal Gestational diabetes mellitus	806 (72.9) 300 (27.1)

IQR is interquartile range.

### Risk factors for GDM

Risk factors for GDM are described in Table 3. The results from multiple logistic regression analysis showed that women aged 25–34 and ≥35 years had higher aORs for GDM compared with those younger than 25 years. The aORs were 2.0 (95%CI 1.3–2.9) and 3.0 (95%CI 1.7–5.4), respectively. Family history of diabetes and a pregestational BMI of 23 kg/m^2^ or higher were also associated with increasing odds of GDM with aORs of 1.9 (95%CI 1.3–2.9) and 1.6 (95%CI 1.1–2.3). Fertility treatment was associated with strong increasing odds of GDM, with a crude OR of 3.3 (95%CI 2.0–5.4) and an aOR of 2.3 (95%CI 1.3–3.8) after adjusting for maternal age, pregestational BMI, family history of diabetes, and parity.

**Table 3.** Risk factors of GDM (*n* = 1,106).

Factors	Total N = 1,106	GDM n (%) N = 300	Crude OR (95%CI)	*Adjusted OR (95%CI)	**Adjusted OR (95%CI)
Living area					
Rural Urban	673 433	168 (25.0) 132 (30.5)	1 1.3 (1.0–1.7)	1 1.2 (0.9–1.6)	1 1.2 (0.9–1.6)
Maternal age					
≤24 25–34 ≥35	307 667 132	50 (16.3) 193 (28.9) 57 (43.2)	1 2.1 (1.5–3.0) 3.9 (2.5–4.2)		1 2.0 (1.3–2.9) 3.0 (1.7–5.4)
Pregestational BMI					
<23 ≥23	925 181	229 (24.8) 71 (39.2)	1 2.0 (1.4–2.7)	1 1.7 (1.2–2.4)	1 1.6 (1.1–2.3)
Family history of diabetes					
No Yes	971 135	245 (25.2) 55 (40.7)	1 2.0 (1.4–3.0)	1 2.0 (1.4–2.9)	1 1.9 (1.3–2.9)
Parity					
0–1 2 +	883 223	221 (25.0) 79 (35.4)	1 1.6 (1.2–2.3)	1 0.9 (0.6–1.3)	1 1.0 (0.7–1.4)
Fertility treatment					
No Yes	1,038 68	264 (25.4) 36 (52.9)	1 3.3 (2.0–5.4)	1 2.5 (1.5–4.1)	1 2.3 (1.3–3.8)
PCOS					
No Yes	1,050 56	279 (26.6) 21 (37.5)	1 1.7 (1.0–2.9)	1 1.7 (1.0–3.1)	1 1.5 (0.8–2.7)
Use of anti-depressive medication					
No Yes	1,098 8	296 (27.0) 4 (50.0)	1 2.7 (0.7–10.9)	1 2.7 (0.6–11.4)	1 1.9 (0.4–8.4)
Exposed to smoke					
No Yes	733 373	207 (28.2) 93 (24.9)	1 0.8 (0.6–1.1)	1 0.9 (0.7–1.2)	1 0.9 (0.7–1.2)
Low-intensity physical activity per week (*n* = 1088) ≤3 h >3 h	788 300	216 (27.4) 79 (26.3)	1 1.0 (0.7–1.3)	1 0.9 (0.6–1.2)	1 0.8 (0.6–1.2)
High-intensity physical activity per week (*n* = 1094)					
≤1 h per week >1 h per week	1,075 19	289 (26.9) 5 (26.3)	1 1.0 (0.4–2.7)	1 1.0 (0.4–3.0)	1 1.1 (0.4–3.1)

GDM is gestational diabetes mellitus; OR is odd ratio; CI is confidence interval; BMI is body mass index; PCOS is polycystic ovary syndrome; IQR is interquartile range.

*Adjusted for maternal age.

**Adjusted for maternal age, pregestational body mass index, family history of diabetes, parity, and fertility treatment.

Among the 645 women with previous delivery (Table 4), a history of GDM and previous macrosomia were associated with an increased risk for GDM, with crude ORs of 2.9 (95%CI 1.4–6.2) and 2.5 (95%CI 1.2–5.3), respectively. After adjusting for maternal age, pregestational BMI, family history of diabetes, fertility treatment, parity, and history of macrosomia, only a history of GDM remained significantly associated with increased risk for GDM with an aOR of 3.1 (95%CI 1.4–6.9). There was a tendency for a history of macrosomia to be associated with increased odds of GDM, though the association was not significant (aOR 2.1; 95%CI 1.0–4.8).

**Table 4.** Associations between obstetric risk factors and GDM among multiparous women (*n* = 645).

Factors	Total N = 645	GDM n (%) N = 191	Crude OR (95%CI)	*Age-adjusted OR (95%CI)	**Adjusted OR (95%CI)
History of GDM					
No Yes	617 28	176 (28.5) 15 (53.6)	1 2.9 (1.4–6.2)	1 3.0 (1.4–6.6)	1 3.1 (1.4–6.9)
History of macrosomia					
No Yes	617 28	177 (28.7) 14 (50.0)	1 2.5 (1.2–5.3)	1 2.3 (1.1–5.1)	1 2.1 (1.0–4.8)
History of pregnancy-induced hypertension					
No Yes	633 12	186 (29.4) 5 (41.7)	1 1.7 (0.5–5.5)	1 1.5 (0.4–4.8)	1 1.4 (0.4–4.7)
History of induced abortion					
No Yes	573 72	165 (28.8) 26 (36.1)	1 1.4 (0.8–2.3)	1 1.3 (0.8–2.2)	1 1.2 (0.7–2.0)
History of spontaneous abortion					
No Yes	577 68	166 (28.8) 25 (36.8)	1 1.4 (0.9–2.4)	1 1.3 (0.7–2.2)	1 1.3 (0.8–2.3)
History of stillbirth					
No Yes	570 75	166 (29.1) 25 (33.3)	1 1.2 (0.7–2.0)	1 1.0 (0.6–1.7)	1 1.0 (0.6–1.7)
History of premature birth					
No Yes	596 49	178 (29.9) 13 (26.5)	1 0.9 (0.4–1.6)	1 0.8 (0.4–1.5)	1 0.8 (0.4–1.7)

GDM is gestational diabetes mellitus; OR is odd ratio; CI is confidence interval.

*Adjusted for maternal age.

**Adjusted for maternal age, pregestational body mass index, family history of diabetes, parity, fertility treatment, history of gestational diabetes, and history of macrosomia.

## Discussion

More than one-fourth (27.1%) of the women in our study had GDM. The risk factors of GDM were advanced maternal age, high pregestational BMI, family history of diabetes, and previous GDM. In addition, fertility treatment was found to be strongly associated with an increased risk of GDM.

In our study, the prevalence of GDM was similar to the prevalence of GDM reported in Thailand (29.2%) [20], but higher than in previous studies in Vietnam, where the prevalence of GDM in metropolitan areas of Vietnam, using the same diagnostic criteria, ranged from 18% to 22.8% [14–16]. The comparatively higher prevalence found in our study is somewhat surprising. However, our findings most likely reflect a trend where women are increasingly changing their lifestyle into more sedentary behaviours and their dietary intake into more processed food and drinks [21]. We found that advanced maternal age, high pregestational BMI, family history of diabetes mellitus, and history of GDM were associated with GDM. These associations are well documented globally as well as in Asia [8,22]. In addition, fertility treatment, including ovulation induction, intrauterine insemination, IVF, and intracytoplasmic sperm injection, was significantly associated with an increased risk of GDM. Despite the widespread and increasing use of fertility treatments in low- and middle-income countries, few studies have assessed the association between fertility treatment and GDM in these settings. However, studies from high-income countries have noted a similar finding regarding the association between fertility treatment and GDM. A recent register-based study from Finland [11] reported an aOR of 1.26 for GDM among women who conceived after IVF compared to those who conceived naturally. Similarly, a systematic review [10] reported an increased relative risk of 1.53 for GDM among women achieving singleton pregnancy by either IVF or intracytoplasmic sperm injection compared with those achieving singleton pregnancy spontaneously. Our data did not allow for a detailed analysis of how different types of fertility treatments are associated with GDM, and the details regarding the underlying mechanistic link between fertility treatment and GDM in our study are yet to be determined. However, it may be related to progesterone, which is used in fertility treatment to enhance embryo implantation and reduce the risk of miscarriage [23]. This assumption is in line with previous studies reporting an increased risk of GDM in women treated with progesterone [24,25]. For example, Waters et al. [26] found that 24% of the women in the progesterone group, compared to 11% in the control group, developed impaired glucose tolerance during pregnancy. This association between progesterone treatment and GDM could be due to increased insulin resistance in skeletal muscle and adipose tissue, potentially resulting from weakened expression of glucose transporter 4 [27,28]. Moreover, women undergoing fertility treatment may have a higher rate of pregestational obesity, advanced maternal age, and PCOS, all of which are risk factors of GDM [29].

In our analyses, we accounted for the effects of maternal age, pregestational BMI, parity, and family history of diabetes on associations between fertility treatment and GDM. However, we did not include PCOS in the adjusted analysis since our data were based on self-reported information from women involved rather than diagnoses confirmed by the Rotterdam criteria which could affect the accuracy and reliability of our analysis [30]. The global prevalence of PCOS, as estimated using these criteria, stands at 11.5% [31]. Hence, the PCOS prevalence of 5.1 found in our study is most likely severely underreported. Since PCOS is linked with an increased risk of both infertility and GDM [32], it may be speculated whether our results reflect a hidden association between PCOS and GDM. However, in Bosdou’s study, a meta-analysis that included subgroup analysis based on whether studies included or excluded women with PCOS, found that this did not change the significance or the direction of the effect observed regarding the type of conception and the presence of GDM. Nonetheless, the relative risk was higher among studies excluding women with PCOS (RR = 4.12) than in those including them (RR = 1.49) [10]. Considering the increasing number of women undergoing fertility treatment in Vietnam and the adverse effects of GDM on both the mother and offspring, doctors and couples need to be aware of the potential association between fertility treatment and GDM. This awareness is increasingly crucial as more Vietnamese couples, for various reasons, opt for fertility treatments [33], even though they may be able to conceive naturally [34]. However, more research is needed to assess more thoroughly the association between fertility treatment and GDM, preferably, studies that also take PCOS into consideration and utilize globally accepted PCOS diagnostic criteria.

### Strengths and limitations

A strength of the present study is that the diagnosis of GDM was obtained using internationally accepted rigorous diagnostic criteria where OGTT was offered to all pregnant women who attended routine antenatal care. Also, women were recruited from both public and private health facilities, a circumstance that adds to the representativity of the study. Another strength is the fairly large sample size, which allowed us to examine factors that are less known to be associated with GDM in settings in lower- and middle-income countries, such as fertility treatment.

The study also has several limitations. Firstly, it was a cross-sectional study, which inherently carries limitations in establishing causality. Secondary, our analysis relied on self-reported data concerning pregestational BMI, fertility treatment, and PCOS. This approach may have introduced recall bias, potentially affecting the accuracy of the data collected. However, when it comes to pregestational BMI and fertility treatment, we believe that Vietnamese women are aware of their weight, and we also believe that women are aware of whether they have undergone fertility treatment or not in relation to their present pregnancy. In contrast, PCOS is not given much attention in Vietnam, and most women are not aware of whether they have PCOS. Thus, the information we obtained about PCOS may not provide the true picture of the problem. Another limitation is that we recruited pregnant women attending antenatal care at two facilities. Although these facilities represent both private and public health providers, it may still have led to selection bias as they are quite large with relatively advanced diagnostic setups, which may have attacted more well-off women. Additionally, both health facilities being located in Thai Binh City may explain the higher proportion of women with tertiary education levels (59.0%) compared to the general population. Finally, we used two different enzymatic methods for measuring venous glucose. Internationally, there is no gold standard for glucose measurement [35], and it has been suggested that different enzymatic methods do not affect the estimated prevalence of diabetes mellitus [36]. However, when focusing on GDM, it is largely unknown how different enzymatic methods may affect prevalence rates. This concern is supported by a GDM study that reported that the prevalence of GDM identified by the hexokinase method was lower than that by the glucose oxidase method, while mean plasma glucose concentrations measured by the hexokinase method were higher [37]. In our study, more than two-thirds of OGTT cases were diagnosed by the hexokinase method, and it may be speculated whether the GDM prevalence is, therefore, underestimated in our study.

## Conclusion

The prevalence of GDM in Vietnam is high, affecting over a quarter of pregnant women. Risk factors include advanced age, overweight, family history of diabetes, prior GDM, and fertility treatment. Our findings highlight the need for enhanced public health initiatives to address GDM in Vietnam. The under-recognition and suboptimal management of GDM can have severe consequences, including increased risks of maternal complications, adverse neonatal outcomes, and long-term health issues such as type 2 diabetes for both mothers and their offspring. To mitigate these risks, we suggest culturally tailored educational campaigns to raise awareness about GDM among women of reproductive age, their families, and healthcare providers. Also, information about GDM should be integrated into routine antenatal care, and targeted screening should be offered for women with identified risk factors, such as obesity, advanced maternal age, or a history of GDM. Finally, women undergoing fertility treatment should be informed about their potential risks for GDM and offered tailored monitoring and management plans.

## Data Availability

The data underlying this article will be shared upon reasonable request to the corresponding author.
